# Oral health behaviour, practices, status and the dental treatment needs among paliyan and pulayan tribes in India

**DOI:** 10.6026/973206300161113

**Published:** 2020-12-31

**Authors:** Srisakthi Dorai Kannan, Meignana Arumugham Indiran, R Pradeep Kumar

**Affiliations:** 1 Saveetha Dental College, Saveetha Institute of Medical and Technical Sciences, Saveetha University, Chennai 77, India

## Abstract

It is of interest to document the The Oral Health heigene data among the paliyan and pulayan tribes in India. The Paliyan and Pulayan tribes inhabit a narrow strip of Western Ghats in the hilly regions of Madurai, Dindigul, Theni, Tirunelveli and Virudhunagar districts of Tamil Nadu. Subjects aged 5yrs and above are used in this study. The WHO assessment form was used to assess the oral health status. Results show that 589 (58%) ignored the oral health problems, 225 (22.2%) relied on self-medications, 142 (14%) consulted a general physician, 35 (3.5%) had visited a dentist and the remaining 23 (2.3%) were dependent upon over the counter medications from a pharmacy. Thus, the prosthetic need was highest among the 55 + yrs age group, 36 (48.6%) required maxillary prosthesis and 21 (28.4%) required mandibular prosthesis.

## Background

When India gained its political independence in 1947,two India's was mentioned the one, under the direct British administration and the other, under the princely states. A third India, which was ignored and remained, unrecognized at the time, was 'Tribal India' living in forests, cut off from the mainstream of social life of this country. The fruits of independence have not somehow been tasted by this neglected society, which is spread over hills, valleys and plains [1,2]. In 1950, the number of tribal communities was 212. This number has increased since then and currently there are 573 communities, which constitute eight percent of the nation's total population, the second largest tribal population in the world after Africa [3,4]. When compared to various tribal communities in Tamil Nadu, Paliyans and Pulayans constitute relatively a small group [5]. Even though the government implemented various development measures for this ethnic group, they are facing many problems such as inadequate housing, non availability of irrigated land, low price for forest products, delay in issuing ration cards, community certificates and poor quality essential commodities were supplied through ration shops situated far away from tribal areas [4]. For the past 15 years the Paliyan and Pulayan tribes have been getting the health care services through mobile medical units and Primary health centers in some places of the districts [3]. Lack of personal hygiene, poor sanitation, absence of health education, poor awareness, lack of proper transport, poor response level and continuation of traditional practices that affect the health seeking behavior are responsible for the ill health in the tribal population [4]. Like all health problems, dental and oral diseases are a product of economic, social, cultural, environmental and behavioral factors and because of the failure to tackle social and material determinants and to incorporate oral health into general health promotion, millions suffer from untraceable tooth ache and poor quality of life and end up with few teeth [6]. Assessment of the oral health status and associated behaviors is an essential part of the process of planning appropriate and acceptable health services and dental health education programs in order to improve the oral health status of this population. We have successfully completed numerous epidemiological and in-vitro studies and trials for the betterment of our community, and every effort has been made by the team to provide database to evidence based practice [7-24]. Hence the aim of the present study is to assess the oral health status, behavior, practices and treatment needs of the Paliyan and Pulayan tribes in India.

## Methodology

### Study design:

A cross sectional study was designed.

### Study area: 

The study was conducted in the tribal hamlets in and around Pachalur and Periyur Village Panchayats, in the lower Palani hills of Western Ghats, Dindigul district in Tamil Nadu. The study areas Pachalur and periyur cover many individual hamlets belonging to various tribal communities, whose life is woven around the forest ecology and forest resources.There are six hamlets in and around Pachalur panchayat and eight around The Periyur panchayat [25].

### Study population:

The indigenous people of the study area are called Paliyar/ Paliyan and Pulayar/Pulayan. They are found in the hilly regions of Madurai, Dindigul, Theni, Tirunelveli and Virudhunagar districts. When compared to various tribal communities in Tamil Nadu, Paliyans and Pulayans constitute relatively a small group. They are placed at 32nd position of the total population of the scheduled tribe in Tamil Nadu [3]. In the Palani hills they are found at an altitude of up to 2200m. Generally they are illiterate and they speak Tamil. Physically they are similar to the Semong of Malaya and other Indian tribal communities. Historically, these tribal communities have survived on their traditional knowledge base. Traditional medicines are the primary healthcare resources for these tribes to protect their health [25].

### Inclusion criteria:

All tribal subjects aged 5 yrs and above in the selected village.

### Exclusion criteria:

Those who were not willing to participate, or terminally ill and mentally compromised, or who were unavailable at home for enrolment in the study even after three consecutive visits to their homes.

### Approval and Informed Consent:

Approval was obtained from the Scientific Review Board (SRB) and Institutional Ethical Committee (IEC) of Saveetha University. Written informed consent was obtained from all the participants. Prior to the start of the survey, permission was obtained from the president of the village panchayat and the Village Administrative Officer.

### Sample size:

The sample size was calculated based on total mean DMT (5.34±7.68) of Bhil tribes of Southern Rajasthan, by Santhosh Kumar et al. (2009) [8]. N = 928, with power 80% and 5% alpha error.

### Sampling:

Multi stage cluster sampling was employed. In the first stage one district was randomly selected from the five districts (Madurai, Dindigul, Theni, Tirunelveli and Virudhunagar) inhabited by Paliyan and Pulayan tribes, in Tamil Nadu. In the second stage village panchayat/s were randomly selected from the eight panchayats inhabited by these tribes. In the third stage all the hamlets inhabited by the tribes were considered as clusters and all the households in the hamlets were examined completely. New clusters / village panchayats were included till the desired sample size was achieved. All individuals aged 5yrs and above were included in the study.

### Survey instrument:

An interviewer administered questionnaire and a WHO assessment form (1997) [26] were used. The questionnaire was designed to elicit information on oral health behaviour and oral hygiene practices among the tribal population. The WHO assessment form was used to assess the oral health status.

### Clinical examination:

Clinical examination was conducted by a single examiner who had been trained through a series of clinical training sessions at the Department of Public Health Dentistry, Saveetha Dental College, Chennai. The intra-examiner reliability was calculated by examining a group of 20 individuals examined per day and the re- examination was carried out at least 30 minutes after the initial examination. The Kappa value for intra-examiner reliability was 0.7. After recording the questionnaire, dental examinations were conducted in supine positions under natural light by means of a mouth mirror and a CPI probe, which conform to World Health Organization (WHO) specifications. Instruments used in oral examination are to be sterilized using autoclave at 121o c for 15 minutes at 15 lbs pressure. Only completely filled forms were considered for analysis.

### Statistical analysis:

Data was entered in Microsoft Excel spreadsheet and analysed using SPSS software (version 15). Descriptive statistics including mean, standard deviation and percentages were used. For all the tests, a p value of <0.05 was considered statistically significant. Kruskal Wallis test and Chi square test were used to test the association and significance of difference between the groups.

## Results:

Among the 1014 study subjects, 463 (45.6%) belonged to Paliyan tribes and 551 (54.4%) belonged to Pulayan tribes. Among the Paliyan tribes, 255(55.1%) were males and 208 (44.9%) were females. Among the Pulayan tribes 307 (55.7%) were males and 244 (44.3%) were females. Oral health behaviour of the study subjects were assessed using a closed ended questionnaire, Among the study subjects, 589(58%) ignored the oral health problems, 225 (22.2%) relied on self medications, 142 (14%) consulted a general physician, 35 (3.5%) had visited a dentist and the remaining 23 (2.3%) were dependent upon over the counter medications from a pharmacy. The self medications used by the study population gave an insight in to the cultural practices adapted by the community, 195 (19.2%) used self medications for tooth ache/jaw ache. 59 (5.70%) used snuff powder, 48 (4.7%) used clove, 33(3.3%) used the sap from kattu amanaku chew stick, 30(3%) used Kadukkai seed powder, 13(1.3%) used a counter irritant (Bhodai pil thailam) and 12 (1.2%) used sambaga milagai for relieving the pain. Both males and females of the tribal community used different forms of tobacco. Among the 216 males who had no adverse oral habits, 91(16.2%) were Paliyan tribes and 125 (22.2%) were Pulayan tribes.Among the 287 females who had no adverse oral habits, 135 (29.9%) were Paliyan tribes and 152 (33.6%) were Pulayan tribes. When the association between ethnicity and tobacco habits were studied, no significant difference was observed between the groups. Next part of the questionnaire covered details on modes and methods used for brushing teeth. It was found that about 40% of both tribes used toothbrush and toothpaste for cleaning their teeth. When enquired about the self perceived oro dental problems, across all age groups, majority of them complained about Dental pain (35.6%) , followed by dental decay (25.2%). On clinical examination, Leukoplakia and Smokers melanosis were the most common lesions observed in the 15 - 55+ years age group. Figure 1(see PDF) depicts the age wise distribution of periodontal health status among the study subjects, and in the 35 plus year age group, everybody had some form of periodontal disease. And when an association between periodontal health and ethnicity was done, it was not significant, implying the periodontal health was at same level in both the tribal groups. Table 1(see PDF) depicts the dental caries experience among the study subjects. The mean DMFT was highest among the 55+ years age group (3.53 ± 4.70), followed by the 5 - 14 years age group (2.10±2.18), in which the decay was the predominant component. A kruskal wallis test was done to check the significance of difference between and within the age groups and it was found to be statistically highly significant, but no such difference was observed between the two ethnic groups. When the prosthetic status was assessed, out of the 1014 subjects studied, only 1 subject had a mandibular prosthesis. Malocclusion was studied using the DAI Index, among the paliyan tribes, 392 (84.7%) had a normal occlusion and 71 (15.3%) had malocclusion. Among the Pulayan tribes, 401 (72.8%) had a normal occlusion and 150 (27.2%) had malocclusion. Age wise dental treatment needs were calculated using Dentition status and treatment needs index. Across the age groups one surface filling (55.3%) and single crown (18.7%) was a major need in 5-14 years age group, two surface filling (51.2%) and pulpal therapy (20.9%) were major need in 15 - 24 years age group, extraction was a major need (45.6%) in 55+ years age group. The treatment needs across age groups was statistically significant but the same between the two tribal groups was not so (Figure 2 - see PDF). The prosthetic need was highest among the 55 + yrs age group, 36 (48.6%) required maxillary prosthesis and 21 (28.4%) required mandibular prosthesis.

## Discussion:

In the developing countries, oral disease data are primarily collected to aid the planning of the health care systems in the respective countries. Epidemiological data from these countries may allow for an improved understanding of the nature of oral diseases and changing disease patterns at the population level. This is due to the fact that such studies are conducted in populations, which so far have had very limited access to formal oral health care. Given that no studies of this nature have ever been conducted formally on the Paliyan and Pulayan tribes, the present study was done to obtain baseline information of the tribe's oral health behaviour, practices, status and the dental treatment needs for future reference in the planning of dental health services for the tribes. Although the Indian Government has rehabilitated the Paliyan and Pulayan tribes, they still retain their semi-primitive lifestyle and practices. Rehabilitation here applies only in terms of better housing and an elementary school for their children. In the past, the tribes had no idea about causation and prevention of disease. The belief in the interference of a supernatural agency was strong in the context of health and disease. The tribes have their own traditional methods and techniques of dental care. They use snuff powder, clove, sap from kattu amanakku chew sticks and many others for relieving dental pain. Sap from the chew stick and juice from semiti kolunthu leaves were used for treating gingivitis. They even use clove and pepper with tamarind to fill the dental cavities. The traditional, religious beliefs of the tribes have been largely influenced and modified recently and they have started to realize the efficacy of scientific methods of treatments and prevention. This is evident by the number of tribes who availed the services of biomedical practitioners. Among the study groups, 14% had consulted a general physician and 3.5% had consulted a dentist for oro dental problems. This was higher when compared to the results of the study on Bhil tribes [6], which puts our study population in a higher plane of acculturation. However 32.3% of the orang asli tribes had visited a dentist. As a price for urbanisation taking place at an explosive pace, these tribes have adopted the behaviour of modern society. About 50% of these tribes have a habit of using some form of tobacco and the difference in the prevalence of adverse oral habits between the ethnic groups was found to be significant statistically (p<0.05). The tobacco usage was less when compared to the Todas (59.4%) in Nilgiris (27) and Kadukurubas (55.2%) in Mysore (28). The habit of snuff dipping was common to all the three study populations. The tobacco related oral mucosal lesions accounted for up to 23.2%. Leukoplakia was the most common lesion and it was higher (10.3%) when compared to Toda tribes [27]. Unlike the findings from the study on Bhil tribes [6], the Paliyan and Pulayan tribes did have traditional and advanced methods of oral hygiene maintenance. Both the ethnic groups used toothbrushes, followed by fingers and chew sticks. The usage of toothbrush was higher among the younger age groups, whereas the usage of chew sticks was prevalent among the older age groups. The overall usage of chew sticks was less when compared to the Iruligas in Karnataka [29 - check with author]. The prevalence of usage of toothbrush was high when compared to Iruligas and less when compared to the Orang asli tribes [28]. The findings on oral hygiene habits must be viewed with caution. The high rate of tooth brush usage may be influenced by factors like, social desirability, whereby the respondents tend to answer questions on dental health and dental health related behaviour in a socially desirable way. Previous studies [6] [30] have shown that people in developing countries usually have limited access to dental care and as a result, poor oral health. This is certainly true for the present study population, for whom the dental health services are virtually nonexistent. The most common oro-dental problem experienced was pain (35.6%), followed by dental decay (6.8%) and bleeding gums (4.4%). The study on Orang asli tribes (28) revealed that 61% had experienced pain and 28% had bleeding gums. This vast difference could be due to the fact that 51.2% of subjects in the current study did not reveal about the oro-dental problems at the time of the survey. In this study, the overall prevalence of periodontal disease was 87.2%, this was in accordance with the periodontal health status of the Kadukurubas [28] and it was in contradiction with the results on Toda tribes (69.84%) [27]. This lower prevalence in Toda tribes could be related to their diet, they ate large amounts of raw vegetables, which would have resulted in natural cleaning. The prevalence of periodontal disease tends to increase with increasing age. Bleeding was most prevalent among the younger age group. It is noted that pathological pockets were the most common problem in subjects aged 35 yrs and above. Calculus was the most commonly affected condition in all the age groups (40.7%). The prevalence of pockets among the Paliyan and Pulayan tribes was found to be 24.3%, similar results were obtained from the study on Iruliga tribes [29 - check with author]. The difference in the periodontal health status between the Paliyan and Pulayan tribes was not significant statistically. The overall dental caries experience in the Paliyan and Pulayan tribes was 62.2%, this was in close proximity to the caries experience of the Toda tribes (53.9%) [29 - check with author]. But was very high when compared to the Kadukurubas (17.8%) [28]. This vast difference could be due to exposure to civilization; consumption of more refined foodstuff, which is a deviation from their primitive dietary habits. In the present study, the mean number of decayed teeth decreased with increasing age, except that there was a slight increase in the 55+ yrs age group. The mean number of missing teeth increased with age. About 5.8% of the population had missing teeth and this could be explained by the practice of self-extraction among the older age groups. The mean number of filled teeth was 0.02±0.14. This lower filled component and higher decay among the Paliyan and Pulayan tribes, reflects the inaccessibility and unavailability to proper dental care. Similar results were observed in the Bhil tribes [6], except for two facts. First, the mean DT showed steady decrease with increasing age and secondly there was no filled component. The mean DMFT among the Paliyan and Pulayan tribes were 1.99 and 1.80 respectively. The difference in the caries experience between the two ethnic groups was not significant statistically. Prevalence of tooth attrition among the Paliyan and Pulayan tribes was 22.7%, this was similar to the attrition patterns in Todas (23.1%) [27], but Kadukuruba tribes exhibited a higher prevalence of attrition (56.67%). According to the authors, the reasons for this higher prevalence could be attributed to the consumption of roots, tubers, raw meat and coarse food. Since information on diet was not analyzed in this study, the role of diet in wasting disease in this population could not be justified. Malocclusion was not a major problem among the Paliyan and Pulayan tribes. The overall prevalence was only 20.8%. The difference in prevalence of malocclusion between the two ethnic groups was not significant statistically. Similar results were obtained in the Toda tribes (20.6%) [27], but it was even lesser in the Kadukurubas (11.54%) [28]. According to Liu.K.et al [31], the reasons for a good occlusion among tribals may be related to transitional timings, sequelae of tooth replacement for the reasons such as low dental caries experience, adequate jaw growth and good genetic background. Analysis of the data so far reveals that oral diseases remain largely untreated in this population. Unlike the results of the study on Todas [27], there was a significant difference in the treatment needs of the Paliyan and Pulayan tribes. The percentage of Paliyan and Pulayan tribes requiring restoration and extraction was 76.3% and 36.5% against 80.2% and 19.2% in the Toda tribes. One surface fillings (40.4%) and two surface fillings (35.9%) were the most required treatment need followed by extractions (36.5%). One surface filling was the greatest treatment needed in the 15-24 yrs age group. Two or more surface filling was mostly required by the older age group, this could be due to higher prevalence of abrasion in these age groups. Very few teeth in the younger age group were indicated for extraction and the highest need for extraction (45.6%) was among the 55+ years age groups. Similar results were obtained from the study on Bhil tribes, except for the fact that extractions formed the major need in the Bhil tribes [6]. The need for prosthesis among the Paliyan tribes was 6.7% and 5.2% in the maxillary and mandibular arches respectively. The need for prosthesis among the Pulayan tribes was 4.0% and 3.0% in the maxillary and mandibular arches respectively. The prosthetic needs in the Paliyan tribe were almost similar to the overall needs in the Kadukurubas (6.8%) [28], but it was higher when compared to the Pulayan tribes. The higher unmet treatment needs in the study population could be attributed to the lack of awareness, lack of motivation and most important of all, the remoteness of these areas. Similar reports were given by Brenan et al [32], who found that geographic remoteness was a barrier in the receipt of dental care in Australia. One of the limitations of the study is that the results could not be generalized over other ethnic groups in India or other countries. The cross sectional nature of the study precludes the ability to draw inferences on causal relationships. The role of diet and culture in the causation of oral disease and its impact on health care service utilization was not focused in this study. Hence, further research is needed to investigate the oral health of various ethnic groups in India.

## Conclusion

Data shows that prosthetic need was highest among the 55 + yrs age group, 36 (48.6%) required maxillary prosthesis and 21 (28.4%) required mandibular prosthesis among the Paliyan And Pulayan Tribes in Tamilnadu India.
